# Supplementary material for: An entropy-initiated coupled-trait ODE framework for modeling longitudinal cohort dynamics
Source: PLoS One. 2026 Mar 19;21(3):e0344090. doi: 10.1371/journal.pone.0344090 (PMC13001968; doi:10.1371/journal.pone.0344090)
Supplement: S1 Appendix — (PDF) [file pone.0344090.s001.pdf]

Supplemental Material for: *An Entropy-initiated Coupled-Trait ODE Framework for Modeling Longitudinal Cohort Dynamics*

Anderson M. Rodriguez
amr28693@uga.edu

A Worked Example: Calculating Shannon Entropy from SATSA Likert Data

To illustrate we calculate an example of Shannon entropy[1] from Likert data; consider the 1984 response distribution (from the SATSA dataset [2]) for the trait *P9 Satisfaction*, where the leftmost data point corresponds to Likert-response choice "1: exactly right" and response options are listed on a gradient from there to the rightmost option, "5: not right at all":

$$x = [76,\ 231,\ 294,\ 611,\ 698]$$

These raw counts correspond to 5-point Likert-scale responses, with higher values indicating more respondents selecting that option. Entropy is computed directly using:

$$H(x) = -\sum_{i=1}^{5} \left(\frac{x_i}{\sum_j x_j} \right) \log_2 \left(\frac{x_i}{\sum_j x_j} \right)$$

$$= -\left(\frac{76}{1910} \log_2 \frac{76}{1910} + \frac{231}{1910} \log_2 \frac{231}{1910} + \frac{294}{1910} \log_2 \frac{294}{1910} + \frac{611}{1910} \log_2 \frac{611}{1910} + \frac{698}{1910} \log_2 \frac{698}{1910} \right)$$

$$\approx 2.11 \text{ bits}$$

This reflects a high level of response dispersion relative to the theoretical maximum of $\log_2(5) \approx 2.32$ bits, which indicates moderate-to-high trait distributional entropy in the year's responses.

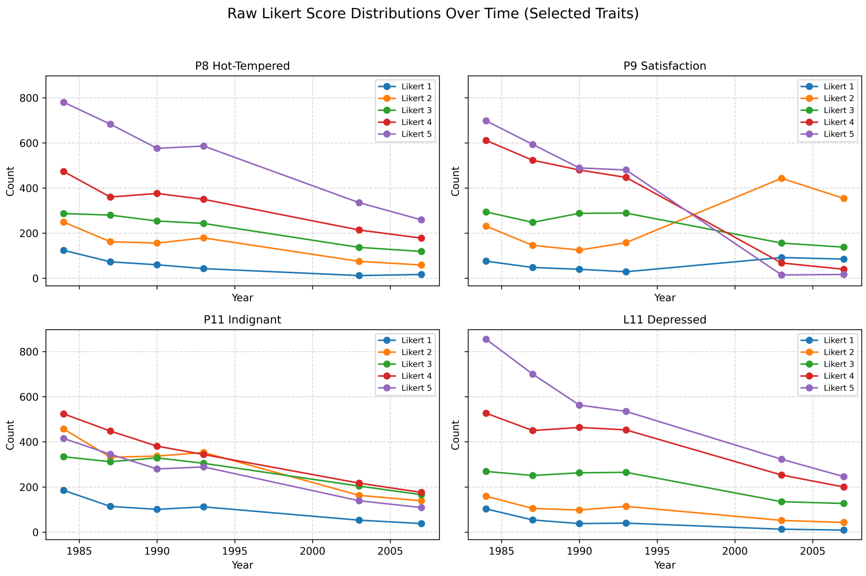

Figure 1: Raw Likert category distributions over time for four of the selected traits: Hot-Tempered, Satisfaction, Indignant, and Depressed traits. Patterns show progressive population convergence for most traits, with Satisfaction showing a divergent spike for the 'Low Satisfaction' response item.

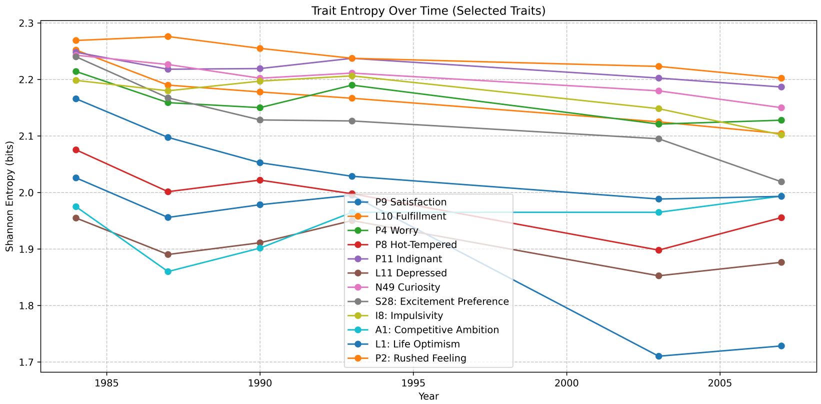

Figure 2: Shannon entropy (in bits) over time for twelve psychometric traits from the SATSA dataset (1984–2007). Most items show gradually declining entropy, reflecting increasingly concentrated response distributions over time.

Appendix B: Entropy Stability Under Simulated Attrition

To test whether observed entropy trends might be artifacts of participant dropout over time, a 50% random attrition rate was applied to all wave-level trait distributions, and Shannon entropy then recomputed for each trait. This simulation is not intended to model the specific mechanisms of real-world attrition, but to serve as a conservative stress test of entropy's sensitivity to substantial random sample loss. Attrition was simulated by randomly reducing the raw count in each Likert response category using a binomial draw: each count x was replaced with $\text{Binomial}(x, 0.5)$, thereby mimicking the effect of 50% random dropout at the individual response level while preserving categorical structure.

This approach approximates individual-level random dropout while preserving the overall response structure, thereby testing whether entropy trends are resilient to cohort attrition. The resulting entropy curves were compared to the original values using root mean square error (RMSE), mean absolute error (MAE), and maximum point-wise divergence [3].

Table 1: Entropy divergence under simulated 50% attrition for all 12 traits. RMSE = Root Mean Square Error; MAE = Mean Absolute Error; Max Diff = largest absolute pointwise difference; Final Δ% = percent change in final-year entropy between original and attrited trajectories.

**Trait**	**RMSE**	**MAE**	**Max Diff**	**% Final Δ**
A1: Competitive Ambition	0.0409	0.0320	0.0806	+4.04
P8: Hot-Tempered	0.0345	0.0205	0.0818	−0.73
P9: Satisfaction	0.0319	0.0225	0.0714	−1.10
L11: Depressed	0.0302	0.0242	0.0623	−3.32
L1: Life Optimism	0.0287	0.0225	0.0537	−2.70
S28: Excitement Preference	0.0243	0.0206	0.0380	+1.79
N49: Curiosity	0.0230	0.0200	0.0355	+1.33
I8: Impulsivity	0.0227	0.0149	0.0513	+0.70
L10: Fulfillment	0.0189	0.0164	0.0333	+0.94
P2: Rushed Feeling	0.0144	0.0132	0.0227	−0.70
P11: Indignant	0.0110	0.0092	0.0194	+0.31
P4: Worry	0.0082	0.0062	0.0164	−0.04

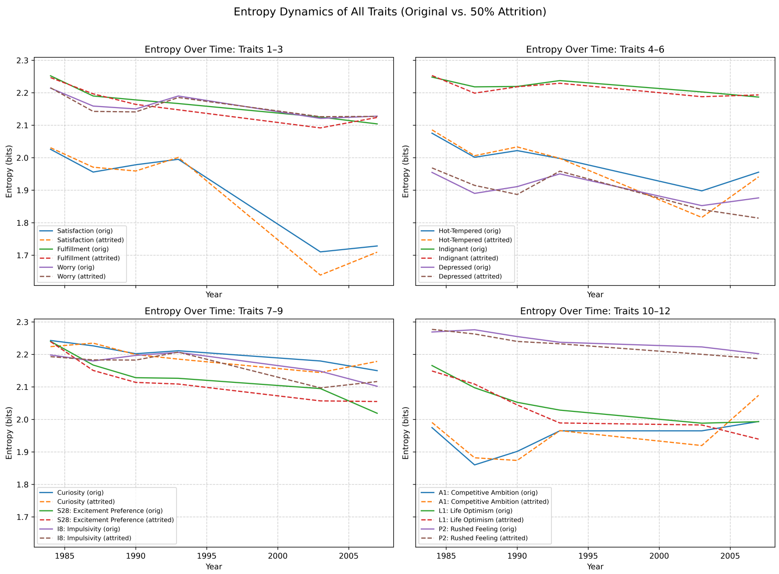

Figure 3: Entropy dynamics after randomized 50% attrition, overlaid with non-attrited results.

Results (Table 1) demonstrate that entropy trajectories remain highly stable under attrition. While visual trends are notable in Figure 3, numerical analysis

demonstrates additional robustness of the entropy summary under substantial sample loss. Across all traits, RMSE values remained below 0.05 bits, with *Worry* and *Indignant* traits showing the greatest robustness (RMSE < 0.012), while *Competitive Ambition* showed the highest divergence (RMSE ≈ 0.041). Percent change in final entropy values ranged between -3.3% and $+4.0\%$, indicating minimal endpoint distortion.

These results suggest that the entropy-based trait dynamics observed in the main analysis are not driven by cohort attrition, but reflect robust features of the population-level response structure. This reinforces the methodological soundness of using entropy as a summary statistic for longitudinal behavioral data, even under substantial sample loss.

Appendix C.1: Model Evaluation: Null vs. ECTO Dynamical Systems

To evaluate whether the ECTO framework captures time-dependent structure beyond trivial alternatives, model performance was benchmarked against simple null baselines that assume no temporal dynamics. For each trait, a flat baseline was constructed by predicting a constant trajectory equal to the empirical mean entropy across all survey waves. This baseline serves as a minimal reference model under sparse longitudinal sampling and provides a conservative comparator for assessing informational gain.

As expected, flat baselines failed to explain variance in the observed entropy trajectories. For the focal traits *Hot-Tempered* (N) and *Worry* (P), constant-mean predictions yielded $R^2 = 0$ by construction and comparatively large RMSE values, confirming that static summaries are insufficient to capture longitudinal structure present in the data.

In contrast, ECTO-based models produced substantially improved fits. The manually specified ECTO variant using normalized entropy recovered major features of the observed trajectories, achieving $R^2 = 0.749$ for *Hot-Tempered* and $R^2 = 0.491$ for *Worry*, alongside marked reductions in RMSE relative to flat baselines. These results indicate that a low-parameter, phenomenological dynamical system can capture nontrivial temporal variation absent from static models.

A globally optimized ECTO variant using raw entropy further reduced pointwise error (RMSE) for both traits. However, for *Hot-Tempered*, this optimization produced predictions with reduced variance, resulting in a strongly negative R^2. This reflects a known limitation of variance-based metrics when model trajectories closely track the mean level but underrepresent temporal variability.

Accordingly, RMSE and R^2 are reported jointly to distinguish pointwise accuracy from variance capture.

figures 4–5 illustrate representative comparisons between flat baselines and ECTO trajectories under raw and normalized entropy formulations. Across formulations, ECTO models consistently outperform static baselines, demonstrating recovery of structured temporal variation in cohort-level entropy that cannot be explained by constant or mean-based models.

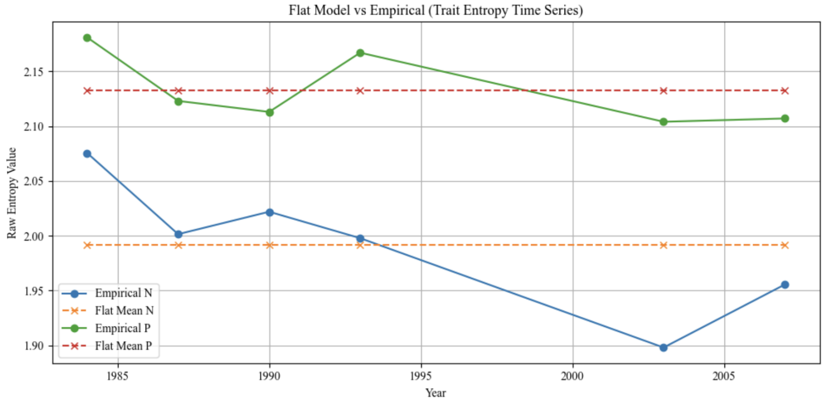

Figure 4: Flat null model fit using raw entropy values for *Hot-Tempered* (N) and *Worry* (P). Both R^2 values were zero, confirming that the flat model lacks temporal explanatory power.

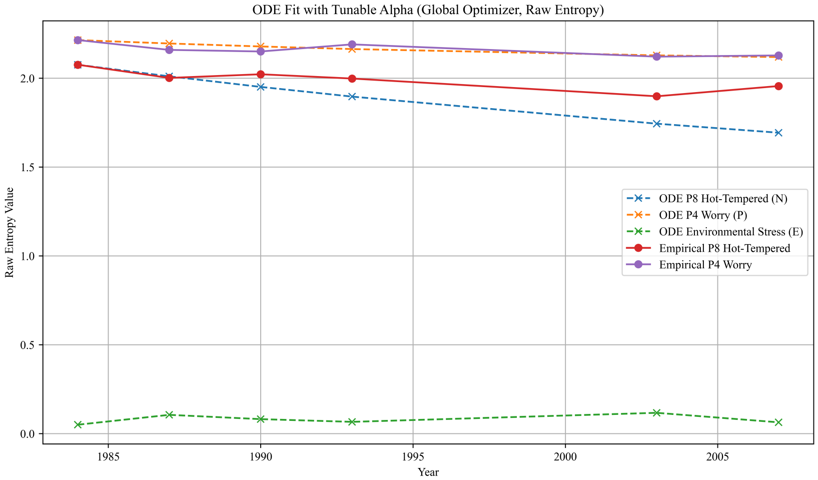

Figure 5: ECTO system with globally optimized parameters and raw entropy input. Although R^2 was poor for *Hot-Tempered* due to low predicted variance, the RMSE and DTW values indicate an accurate match to the empirical trajectory.

Table 2: Comparison of flat null models and ECTO dynamical models for *Hot-Tempered* (N) and *Worry* (P). RMSE and R^2 quantify model fit. Bolded values indicate best performance on each metric, except when negative R^2 indicates over-smoothed or non-informative dynamics (see Repository: *Modules* A_{10} *and* A_{11}).

**Model**	**Entropy Type**	**Trait**	**RMSE**	R^2
Flat Model (Norm)	Normalized	Hot-Tempered (N)	0.3098	0.0000
		Worry (P)	0.3528	0.0000
Flat Model (Raw)	Raw	Hot-Tempered (N)	**0.0550**	0.0000
		Worry (P)	0.0302	0.0000
ECTO Manual	Normalized	Hot-Tempered (N)	0.1552	**0.7490**
		Worry (P)	0.2516	0.4914
ECTO Optimized	Raw	Hot-Tempered (N)	0.134	-4.962
		Worry (P)	**0.022**	**0.550**

Appendix C.2: Parameter Sensitivity

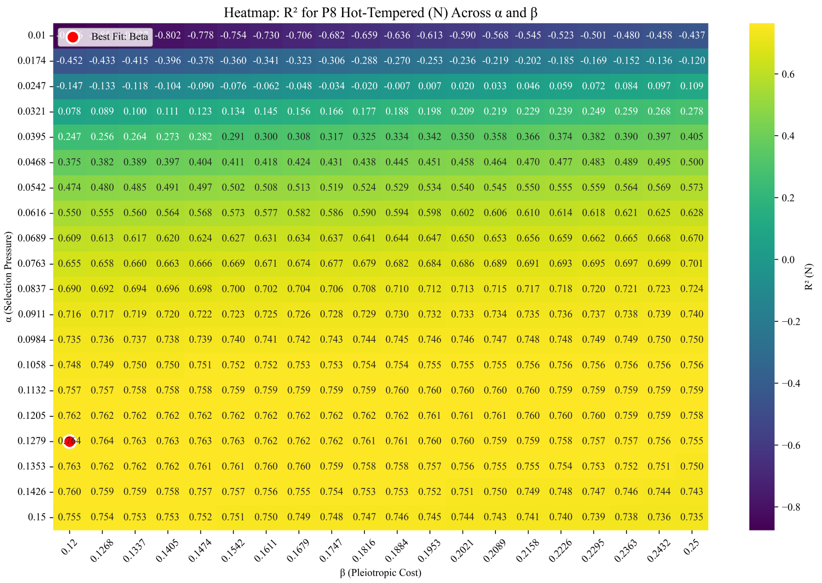

Figure 6: Heatmap of R^2 values for the *Hot-Tempered* trait (N) across a grid of selection pressure (α) and pleiotropic cost (β) values. Each cell represents the R^2 value between simulated entropy and empirical entropy at a given (α, β) pair. The highest-performing configuration is $R^2 = 0.764$ at $(\alpha = 0.128, \beta = 0.120)$.

To assess the sensitivity of ECTO model performance to variation in key parameters, a two-dimensional parameter sweep was conducted over α (selection cost) and β (pleiotropic energy burden), while holding all other parameters fixed. For each of the 400 (α, β) combinations evaluated, entropy trajectories were simulated and compared to the empirical entropy curves using R^2 and RMSE. This analysis is intended as a robustness check, not an optimization procedure, and evaluates whether model performance depends on narrowly tuned parameter choices.

For the primary trait N (*Hot-Tempered*), the sweep revealed a broad region of strong model alignment. Across all parameter combinations, R^2 values ranged from -0.876 to 0.764, with a mean of 0.495 ± 0.391 (SD). A total of 276 out

of 400 (α, β) combinations (69.0%) exceeded the threshold $R^2 > 0.5$. The highest-performing configuration occurred at $\alpha = 0.128$ and $\beta = 0.120$, yielding $R^2 = 0.764$. These results indicate that model performance for N is not confined to a narrow region of parameter space, but persists across a wide range of parameter values.

Figure 6 visualizes the distribution of R^2 values across the (α, β) grid for *Hot-Tempered*. Rather than a single isolated optimum, the heatmap exhibits an extended region of elevated fit quality, with performance degrading gradually outside this region. This pattern supports the conclusion that the observed model alignment is not a consequence of finely tuned parameters.

A corresponding parameter sweep was conducted for the pleiotropic trait P (*Worry*). In contrast to N, R^2 values for P spanned a narrower range, from 0.316 to 0.596, with a mean of 0.451 ± 0.080. Only 127 parameter combinations (31.8%) exceeded $R^2 > 0.5$. This reduced sensitivity to (α, β) variation is consistent with the model formulation in which P is influenced indirectly through coupling and feedback rather than being directly governed by the swept parameters.

Throughout this analysis, the parameter c_3, which weights the contribution of environmental stress within the aggregated constraint term, was held constant at $c_3 = 5.6$ in order to isolate the effects of α and β. Environmental stress itself remains time-varying through its own state equation. In this appendix, an exploratory forced variant was used in which $E_{\text{stress}}(t)$ includes a fixed periodic perturbation,

$$\frac{dE_{\text{str}}}{dt} = \gamma E_{\text{str}} \left(\frac{N}{N+K} \right) + \frac{1}{2} A \sin(\omega t),$$

with amplitude and frequency held constant across all runs. Because this term is fixed across the parameter sweep, variation in model performance can be attributed to changes in (α, β) rather than to differences in environmental forcing.

Taken together, this parameter sweep demonstrates that ECTO model performance for the focal trait N is robust across a substantial region of parameter space, while performance for P is comparatively less sensitive to direct parameter variation. These results reduce the likelihood that reported fits arise from narrow parameter tuning and support the use of a low-dimensional, phenomenological parameterization for exploratory modeling of cohort-level entropy dynamics.

Appendix D: Metabolic Capacity Sensitivity Sweep

In the main model, $G = 1.0$ was chosen to provide an interpretable baseline.

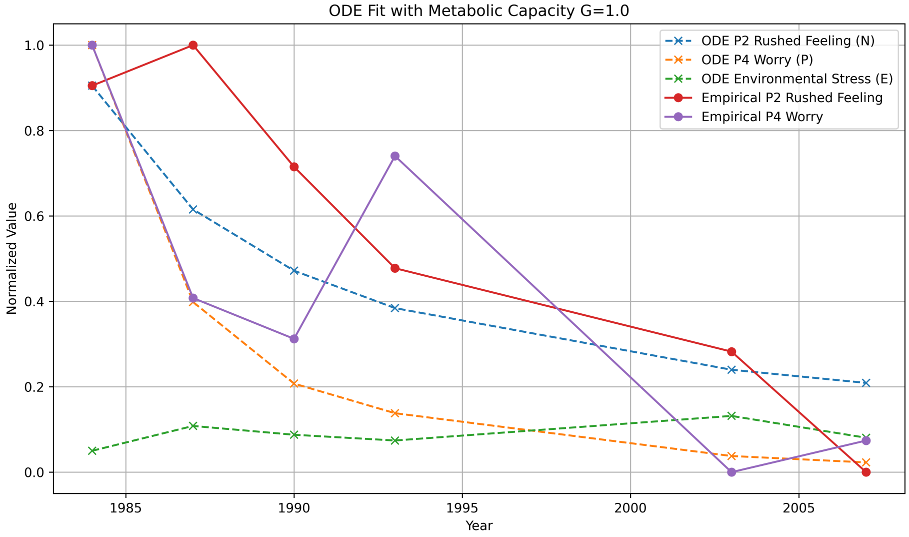

Figure 7: ODE fit with metabolic capacity $G = 1.0$. This parameter set approaches an optimal balance between metabolic supply and trait trajectory.

$$E_{\text{metabolic}} = c_1 P + c_2 N + c_3 E_{\text{stress}}$$

A more extreme constraint was tested at $G = 0.12$.

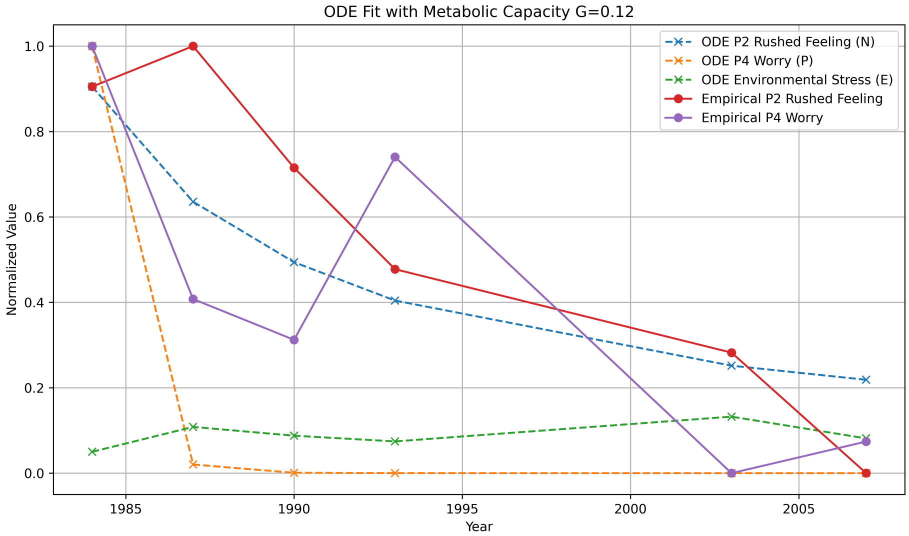

Figure 8: Example model behavior at metabolic capacity $G = 0.12$. This low-G configuration illustrates a boundary case where energy constraints strongly limit trait expression and model–data alignment.

At the upper bound of the spectrum tested, $G = 1.48$ introduced abundant metabolic capacity.

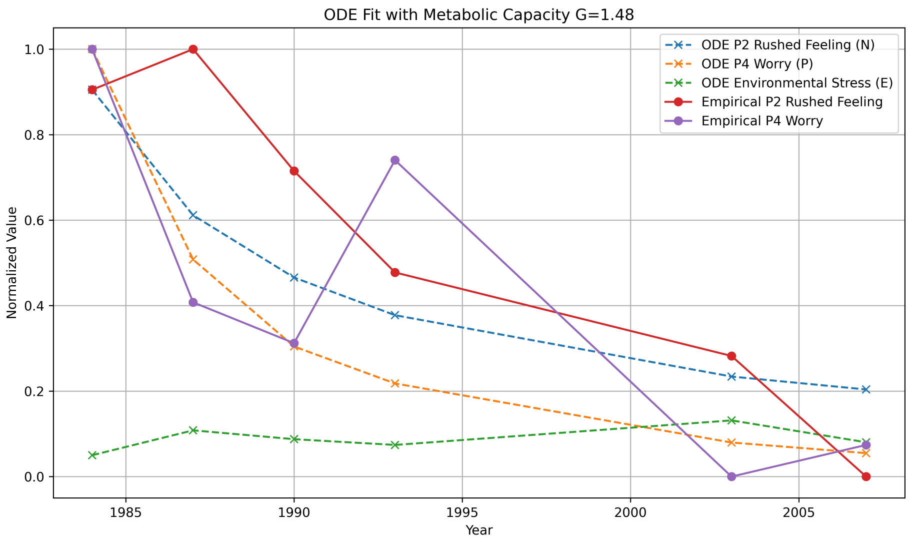

Figure 9: High-capacity condition with $G = 1.48$. This exploratory case reveals that the ODE (P) term has now 'overtightened'.

Table 3: Sensitivity of Model Dynamics to Metabolic Capacity (G)

G	RMSE_N	RMSE_P	R²_N	R²_P	Pearson_r_N	Pearson_r_P	DTW_N	DTW_P
0.1200	0.1981	0.3501	0.6795	0.2036	0.8790	0.7180	0.5160	1.1610
0.4800	0.2043	0.3186	0.6591	0.3406	0.8700	0.7190	0.5270	0.8870
0.9500	0.2083	0.2891	0.6456	0.4569	0.8650	0.7180	0.5360	0.9420
1.0000	0.2086	0.2873	0.6446	0.4636	0.8650	0.7170	0.5380	0.9460
1.4800	0.2108	0.2800	0.6372	0.4908	0.8630	0.7100	0.5540	1.1160

To examine the sensitivity of ECTO model behavior to energetic constraint, a univariate parameter sweep was conducted over the metabolic capacity term G, while holding all other parameters fixed (see: *Appendix D*). This analysis isolates the role of the capacity scaling term in modulating model trajectories and serves as a robustness check on how constraint enters the coupled system.

In the main analyses, $G = 1.0$ was selected as a normalized reference value, allowing energetic costs to be interpreted relative to a unit baseline. At this setting, the system produces balanced trajectories for both the primary trait N (*Hot-Tempered* or *Rushed Feeling*, depending on the run) and the pleiotropic trait P (*Worry*), without introducing strong saturation effects.

Lower values of G impose increasingly stringent capacity constraints on the cost term. At $G = 0.12$, the model produces trajectories in which the primary trait N remains well-aligned with the empirical entropy curve, while the pleiotropic trait P exhibits degraded alignment. This behavior arises directly from the normalization of the aggregated cost by G and does not require any additional parameter adjustments.

At intermediate values (e.g., $G = 0.48$ and $G = 0.95$), N retains stable alignment with empirical data, while P shows gradual suppression. These intermediate regimes illustrate that the model responds smoothly to changes in capacity, with no abrupt transitions or instabilities observed across the tested range.

At higher capacity ($G = 1.48$), the pleiotropic trait P shows improved align-

ment, while N exhibits modest tradeoffs in fit quality. This reflects the redistribution of constraint within the coupled system when energetic limitation is relaxed.

Table 3 summarizes fit metrics across the tested values of G. Across the sweep, model behavior varies systematically with the capacity parameter, indicating that observed changes in trajectory shape are attributable to controlled variation in G rather than parameter retuning or external forcing.

Overall, this analysis demonstrates that the ECTO system responds coherently to variation in the capacity parameter, producing graded changes in trait trajectories under fixed model structure. The results support the internal consistency of the cost–capacity formulation and illustrate how constraint modulation influences model behavior within a low-dimensional, phenomenological framework, which was also demonstrated in the repository associated with this supplemental material, it's associated main manuscript, and the additional data analyzed from the 2025 dataset by Leite et al[4].

References

[1] Shannon, C. E. (1948). A mathematical theory of communication. Bell System Technical Journal, 27(3), 379–423.

[2] Pedersen, Nancy L. Swedish Adoption/Twin Study on Aging (SATSA), 1984, 1987, 1990, 1993, 2004, 2007, and 2010. Inter-university Consortium for Political and Social Research [distributor], 2015-05-13. 10.1017/s0001566000006681

[3] Ray, WJ. (2008). *Methods: Toward a Science of Behavior and Experience* (9th ed.). Wadsworth Publishing.

[4] Leite, T. C., Wankiiri-Hale, C. R., Shah, N. H., Vasquez, C. S., Pavlowski, E. M., Koury, S. E., Kim, J., Ceravolo, K. M., Weinberg, S. M., & Horvath, Z. (2025). Change is never easy: Exploring the transition from undergraduate to dental student in a U.S.-based program. *PLoS ONE*, 20(4), e0321494. 10.1371/journal.pone.0321494

[5] Jaynes, E. T. (1957). Information theory and statistical mechanics. *Physical Review*, 106(4), 620–630.

[6] Kluyver, T., Ragan-Kelley, B., Pérez, F., Granger, B. E., Bussonnier, M., Frederic, J., ... & Willing, C. (2016). Jupyter Notebooks – a publishing format for reproducible computational workflows. In F. Loizides & B. Schmidt (Eds.), *Positioning and Power in Academic Publishing: Players, Agents and Agendas* (pp. 87-90). IOS Press. 10.3233/978-1-61499-649-1-87

[7] McKinney, W. (2010). Data Structures for Statistical Computing in Python. *Proceedings of the 9th Python in Science Conference*, 56-61. 10.25080/Majora-92bf1922-00a

[8] Harris, C.R., Millman, K.J., van der Walt, S.J. et al. Array programming with NumPy. *Nature* **585**, 357–362 (2020). 10.1038/s41586-020-2649-2

[9] Hunter, J.D. (2007). *Matplotlib: A 2D Graphics Environment.* Computing in Science & Engineering, **9**(3), 90-95. 10.1109/MCSE.2007.55
