# Supplementary material for: An entropy-initiated coupled-trait ODE framework for modeling longitudinal cohort dynamics
Source: PLoS One. 2026 Mar 19;21(3):e0344090. doi: 10.1371/journal.pone.0344090 (PMC13001968; doi:10.1371/journal.pone.0344090)
Supplement: Attachment — Submitted filename: Response_to_Reviewers.pdf [file pone.0344090.s002.pdf]

Response to Reviewers

I sincerely thank the Academic Editor and both reviewers for their careful evaluation of the manuscript and for their constructive and detailed feedback. I have revised the manuscript extensively to address all concerns, and in turn have hopefully clarified the scope and interpretation of the proposed framework, and strengthened validation and robustness analyses. Below, I respond point-by-point to each comment raised by the Academic Editor and reviewers, with references to specific sections of the revised manuscript and appendix.

Reviewers' Responses to Editorial Questions

1. Is the manuscript technically sound, and do the data support the conclusions?
Reviewer #1: Partly *Reviewer #2: Partly*
The manuscript has been substantially revised to strengthen the technical grounding and empirical support for the conclusions. Key revisions include the addition of an independent validation dataset (a U.S. dental student longitudinal cohort) to assess generalizability beyond the SATSA dataset (Sections 4.5–4.6), the implementation of leave-one-wave-out (LOO) out-of-sample validation for both datasets (Sections 4.3 and 4.6), and expanded robustness analyses including multi-start optimization, parameter sensitivity sweeps, null model comparisons, and attrition stress tests (Sections 4.7–4.8; Appendix B–D). Several exploratory analyses (marked-up in green font in track-changes-manuscript) have been moved to the appendix or explicitly framed as illustrative rather than definitive.

2. Has the statistical analysis been performed appropriately and rigorously? *Reviewer #1: Yes* *Reviewer #2: No*
To address concerns regarding statistical rigor, I have added explicit out-of-sample forecasting via leave-one-wave-out validation for both datasets (Sections 4.3 and 4.6). The coupled ECTO system is now benchmarked against baseline and null models, including uncoupled dynamics and flat entropy controls (Section 4.4; Appendix C). Parameter identifiability and sensitivity are examined through multistart fitting and parameter sweep analyses (Section 4.7; Appendix C–D), and the limitations imposed by sparse longitudinal sampling are discussed explicitly in the Discussion (Section 5).

3. Have the authors made all data underlying the findings fully available? *Reviewer #1: Yes* *Reviewer #2: Yes*
All data underlying the findings are made available in accordance with PLOS ONE data policy. Publicly available datasets are cited and linked, and all preprocessing, analysis, and simulation code required to reproduce the results is provided in the accompanying repository. Any restrictions associated with third-party data sources (e.g., ICPSR-hosted data) are described explicitly in the Data Availability Statement.

4. Is the manuscript presented in an intelligible fashion and written in standard English? *Reviewer #1: Yes* *Reviewer #2: Yes*
The manuscript has been edited for clarity, concision, and consistency. The main text has been substantially condensed, with secondary analyses and methodological details moved to the appendix.

Reviewer Comments to Author

Reviewer #1

Comment (Paraphrased): The reviewer recommends conducting a limited validation study on a dataset similar to the SATSA cohort to assess generalizability and suggests discussing potential limitations on the types of data applicable to the proposed framework.

Response: I have addressed this comment in two ways. First, I have added an independent validation analysis using a longitudinal U.S. dental student cohort, which differs from SATSA in population, measurement context, and survey structure. This dataset is analyzed using the same entropy preprocessing and ECTO modeling pipeline, with separate parameter fitting and leave-one-wave-out validation (Sections 4.5–4.6). The inclusion of this cohort provides an external test of the framework beyond the original aging twin dataset and demonstrates that the proposed approach is not specific to SATSA.

Second, I have expanded the discussion of scope and limitations to clarify the types of longitudinal psychometric data for which the framework is appropriate. In particular, I emphasize that the method is intended for sparsely sampled cohort-level data with repeated categorical measurements, and that it is not designed to recover individual-level dynamics or mechanistic biological processes (Section 5). These clarifications are intended to delimit the domain of applicability and avoid overgeneralization.

Reviewer #2

Comment (Paraphrased): The reviewer raises concerns regarding the definition and interpretation of neuroticism, the interpretation of the ODE terms, the role and meaning of entropy, parameter identifiability under sparse sampling, statistical validation and forecasting, claims regarding attractors and Lyapunov-like metrics, baseline comparisons, the definition of environment, repository documentation, and manuscript length.

Response: I address these points as follows.

Definition of neuroticism: The Introduction now explicitly defines neuroticism as a psychometric construct derived from questionnaire-based composite measures, rather than as a mechanistic biological entity. This clarification is stated at the outset to avoid causal or biological misinterpretation (Section 1).

Interpretation of the ODE terms: All state variables and parameters are now defined explicitly. Terms such as selection pressure, pleiotropy, metabolic constraint, and environmental feedback are described as phenomenological analogues rather than mechanistic claims. The manuscript states explicitly that these labels are interpretive aids and not direct biological mappings (Section 3).

Role and interpretation of entropy: Entropy is now consistently framed as a population-level summary statistic computed from Likert response distribution measured in waves, and used solely to initialize the dynamical system. Entropy is not treated as a time-varying causal driver. Claims implying causal interpretation have been softened or removed (Sections 2.1–2.4; Section 5; Appendix A).

Units and scaling: Parameter units and scaling are described explicitly, and the system is presented in dimensionless form following normalization. The implications of irregular temporal sampling and nondimensionalization are discussed in the Methods (Section 3).

Parameter identifiability and sensitivity: Given the limited number of timepoints, I have added explicit sensitivity analyses. These include multistart optimization, two-dimensional parameter sweeps, and univariate capacity sweeps, which demonstrate that model behavior is not confined to narrowly tuned parameter values (Section 4.7; Appendix C & D). Limitations due to sparse sampling are discussed explicitly (Section 5).

Statistical validation and forecasting: Leave-one-wave-out validation has been implemented for both the SATSA and dental student datasets to assess out-of-sample predictive performance (Sections 4.3 and 4.6).

Attractor and Lyapunov-related claims: Claims regarding attractor structure and Lyapunov-like behavior have been substantially softened. Exploratory analyses related to these concepts have been moved to the appendix and are framed as illustrative rather than definitive. No strong claims of multistability or rigorous dynamical invariants are made in the main text (Section 5; Appendix C).

Baseline comparisons: The coupled ECTO system is benchmarked against simpler alternatives, including uncoupled dynamics and flat entropy baselines. These comparisons are reported explicitly using RMSE and R^2, with limitations of variance-based metrics discussed where appropriate (Section 4.4; Appendix C.1).

Definition of environment: The environmental term is now defined explicitly as an aggregated constraint state within the phenomenological model, rather than as a measured external variable. Its role and limitations are clarified to avoid misinterpretation as an empirically observed environmental signal (Section 3).

Reproducibility and repository structure: The repository has been aligned with the documented analysis pipeline. Preprocessing steps, analysis scripts, and dependencies are clearly labeled, and environment specifications have been added to support reproducibility. The repository now has a 'A' and 'B' tracks, each of which explore a different dataset, respectively.

Manuscript length and scope: The manuscript has been substantially condensed. Secondary analyses, exploratory extensions, and theoretical elaborations have been moved to the supplemental appendices, and the main text now focuses on the core methodological contribution and primary validation results.
